# Beyond cell replacement: unresolved roles of NG2-expressing progenitors

**DOI:** 10.3389/fnins.2014.00122

**Published:** 2014-05-23

**Authors:** Enrica Boda, Annalisa Buffo

**Affiliations:** Department of Neuroscience Rita Levi-Montalcini, Neuroscience Institute Cavalieri Ottolenghi, University of TurinTurin, Italy

**Keywords:** buffering, depolarization, myelin, neuroprotection, neuromodulation

## Abstract

NG2-expressing parenchymal precursors (NG2+p) serve as primary source of myelinating oligodendrocytes in both the developing and adult Central Nervous System (CNS). However, their abundance, limited differentiation potential at adult stages along with stereotypic reaction to injury independent of the extent of myelin loss suggest that NG2+p exert functions additional to myelin production. In support of this view, NG2+p express a complex battery of molecules known to exert neuromodulatory and neuroprotective functions. Further, they establish intimate physical associations with the other CNS cell types, receive functional synaptic contacts and possess ion channels apt to constantly sense the electrical activity of surrounding neurons. These latter features could endow NG2+p with the capability to affect neuronal functions with potential homeostatic outcomes. Here we summarize and discuss current evidence favoring the view that NG2+p can participate in circuit formation, modulate neuronal activity and survival in the healthy and injured CNS, and propose perspectives for studies that may complete our understanding of NG2+p roles in physiology and pathology.

## Introduction

During Central Nervous System (CNS) ontogenesis, myelinating oligodendrocytes originate from highly ramified neural precursors expressing the platelet-derived growth factor alpha receptor (PDGFRa) and the NG2 chondroitin sulfate proteoglycan (Zhu et al., 2008a,b). These precursors persist in the adult CNS parenchyma, where constitute the main proliferative cell type and make up about 5% of all CNS cells (Dawson et al., 2003). At adult stages, they can engage into maturation to sustain a certain degree of basal myelin turnover and plasticity (Wang and Young, 2013; Young et al., 2013) and are rapidly mobilized to replace oligodendrocytes in demyelination (Redwine and Armstrong, 1998; Zawadzka et al., 2010). Herein we will refer to these cells as NG2-expressing precursors (NG2+p), although NG2 is also expressed by pericytes of the vasculature (Stallcup, 2002) and reported in astrocyte subsets (Matthias et al., 2003).

Currently, the only unequivocally established functions of NG2+p are to regenerate themselves and produce oligodendrocytes in the healthy, diseased and aged CNS (Zhu et al., 2008a,b; Kang et al., 2010; Tripathi et al., 2010; Young et al., 2013). However, the persistence of a large pool of quiescent (i.e., neither engaged in proliferation nor maturation) NG2+p with limited differentiation potential at adult ages has suggested that these cells do not only represent a transitional stage along the oligodendroglial lineage, but rather a novel type of glia endowed with specific properties and functions (Nishiyama et al., 2002). Consistently, NG2+p appear uniformly distributed in the gray and white matter and provide a stereotypic reaction to injury independently of the extent of myelin loss, suggesting that they may play roles additional to myelin production. Along this line, it has been proposed that NG2+p may be multipotent progenitors endowed with the ability to generate astrocytes and neurons in defined conditions. However, to date consensus is established only for the generation of astrocyte subsets at perinatal stages (Rivers et al., 2008; Zhu et al., 2008a,b; Huang et al., 2014). Here we will overview and discuss features potentially related to surveillance, neuromodulation and neuroprotection that still render NG2+p very enigmatic and prompt further investigations on this type of glia.

## Ng2+p anatomical relationship with cns cells and paracrine interactions

In both human and rodent CNS, NG2+p appear distributed rather homogeneously in gray and white matter areas, with no correlation between their density and that of myelin (Butt et al., 2005; Staugaitis and Trapp, 2009). NG2+p processes extend tridimensionally to cover non-overlapping fields that are likely maintained by homotypic repulsive mechanisms (Hughes et al., 2013). Accordingly, in the intact tissue contacts among NG2+p processes are rarely observed (Hughes et al., 2013) and, despite some NG2+p express connexin 32 (Melanson-Drapeau et al., 2003), cells are never coupled via gap-junctions (Wallraff et al., 2004; Butt et al., 2005). Thus, at variance with astrocytes, NG2+p do not function as a syncythium, but are rather individual functional units. However, they partly couple to mature oligodendrocytes, indicating some privileged communication with other elements in the lineage (Maglione et al., 2010).

**Figure 1:**
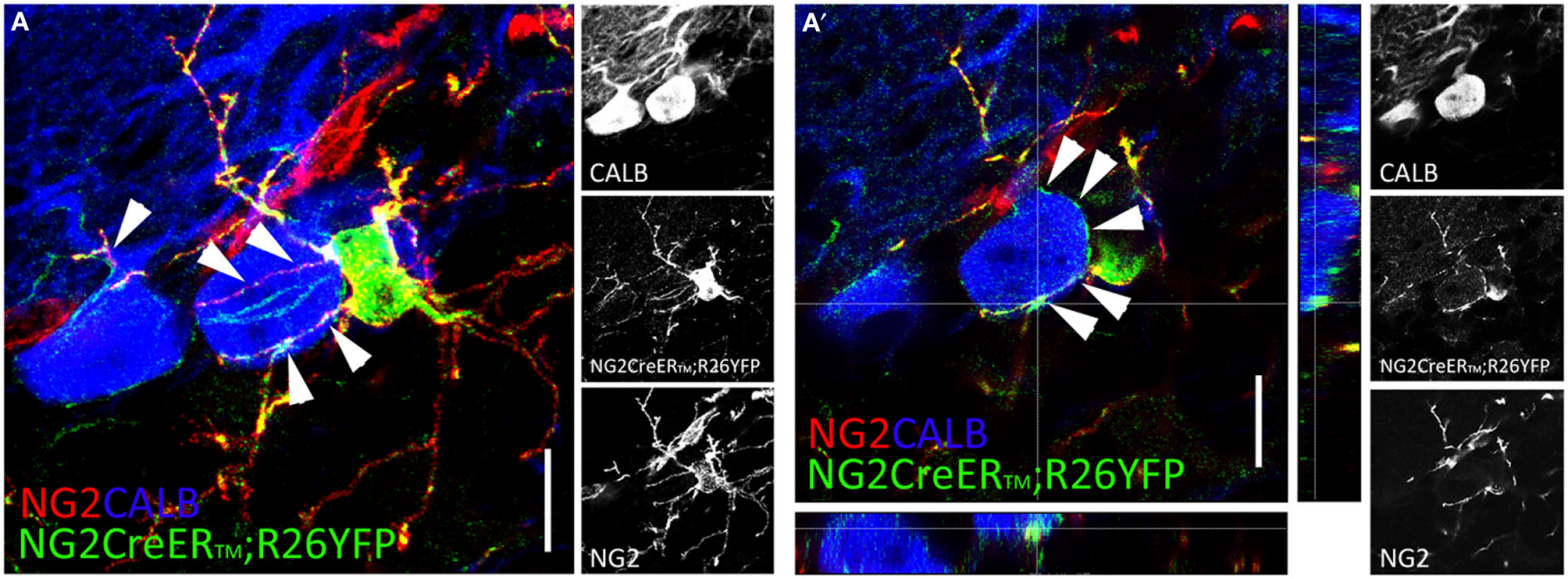
**NG2+p distribution in the adult cerebellum. (A,A′)** Two weeks after tamoxifen induction, multiprocessed cells identified by NG2 (red), and YFP (green) expression appear closely associated to calbindin+ (blue) Purkinje cells in adult NG2CreER™; R26YFP mouse cerebellum. NG2+p arborizations envelop Purkinje cell somata and dendrites [arrowheads in **(A,A′)**]. **(A)** Confocal stack comprising 20 optical section 1 mm thick. **(A′)** Single confocal plane. Scale bars: 10 μm.

Confocal and electron microscopy analyses showed that NG2+p establish intimate anatomical and functional contacts with other CNS cells. NG2+p processes form multiple contacts with dendrites and axons, and NG2+p arborizations intertwine amongst and can encapsulate neuronal somata (Wigley and Butt, 2009; see also Figures 1A,A′) in ways suggestive of their participation in perineuronal nets (Butt et al., 2005). Electron microscopy further revealed that NG2+p processes make contacts with the axonal membrane at the paranodes and nodes of Ranvier (Butt et al., 1999), and interdigitate between pre- and post-synaptic neuronal elements (Ong and Levine, 1999). Contacts with axons include also functional neuron-to-NG2+p synapses (see below). Of note, tight NG2+p-neuron associations exist also in the CNS of adult non-mammalian vertebrates (i.e., zebrafish; März et al., 2010), in line with a fundamental mechanism of communication conserved through species.

Intimate physical interactions also occur with astrocytes. Both cell types often contact the same neurons/axonal terminals (Wigley and Butt, 2009) but NG2+p never ensheat neuronal synapses as astrocytes do. Interestingly, areas of immediate apposition in NG2+p and astrocyte processes are sites of communication, where astrocyte-derived signals induce Ca^2+^ transients in NG2+p (Hamilton et al., 2010). At those sites synaptophysin-positive clusters indicative of secretory vesicle accumulation were observed in NG2+p, pointing to potential secretory spots (Wigley and Butt, 2009). NG2+p also make direct contacts with microglia (Nishiyama et al., 1997), pericytes and myelin (Butt et al., 2005). Hence, NG2+p connect to distinct cell types and functionally-relevant cellular domains, suggesting that they may actively sense and integrate information from diverse sources. An additional level of integration occurs through paracrine signals produced by neighboring cells including neurons, astrocytes and microglia, and influences NG2+p during developmental myelination and in pathology (see Clemente et al., 2013 for review). Is the output of this integration (see below) limited to the regulation of NG2+p differentiation or survival? It is surprising that NG2+p are generally considered only as a sink and not as a source of signals. Hence, if and how NG2+p affect surrounding cells remains essentially unknown.

## Privileged contacts with neurons

What makes NG2+p unique amongst other glial cells is their connection with neurons through synapses that sense neuronal activity at the quantal level with high temporal and spatial resolution. These contacts emerge in parallel with neuronal synaptogenesis and appear ubiquitary, being present in all regions examined so far (cerebellar and cerebral cortex, Lin et al., 2005; Kukley et al., 2008; Ge et al., 2009; Tanaka et al., 2009; Vélez-Fort et al., 2010; hippocampus, Lin and Bergles, 2004; Mangin et al., 2008; brain stem, Müller et al., 2009; white matter tracts, Kukley et al., 2007; Ziskin et al., 2007; Káradóttir et al., 2008; De Biase et al., 2010; Etxeberria et al., 2010). Synapses include glutamatergic and Gamma-Aminobutyric Acid (GABA)-ergic inputs, and both produce depolarizations. Full functionality of these contacts *in vivo* is attested by recordings of evoked, spontaneous, and miniature currents both in physiology and during remyelination (Etxeberria et al., 2010; Vélez-Fort et al., 2010). Glutamatergic contacts are lost as NG2+p progress in differentiation (De Biase et al., 2010; Kukley et al., 2010), in line with a role in the regulation of the cell cycle or of functions specific of the progenitor stage. Notably, glutamatergic inputs increase in frequency and amplitude in NG2+p cells during CNS maturation (Mangin et al., 2008), whereas in the cerebral cortex GABAergic signaling shifts from activation of synaptic receptors to indirect activation of extrasynaptic channels through spillover (Vélez-Fort et al., 2010; Balia et al., 2013; Passlick et al., 2013).

Glutamate- and GABA-induced depolarizations in NG2+p are overall modest in amplitude with the notable exception of cerebellar climbing fiber inputs that induce relevant potential variations (Lin et al., 2005). Thus, to affect the cell physiology, a number of convergent inputs from diverse synapses likely require be integrated. Transduction of synaptic signal may also rely on calcium-mediated mechanisms such as calcium entry though permeable α-amino-3-hydroxy-5-methyl-4-isoxazolepropionic acid (AMPA) or N-methyl-D-aspartate (NMDA) receptors, and activation of voltage dependent conductances that provide signal amplification and can trigger calcium transients from intracellular stores. Notably, in the hippocampus neuron-to-NG2+p synapses undergo activity-dependent modifications analogous to long term potentiation (LTP) at excitatory synapses (Ge et al., 2006), showing that these contacts possess the machinery to sustain plastic changes. Moreover, glutamate or GABA evoked signals can be integrated intracellularly with responses to other mediators such as adenosine triphosphate (ATP), which, upon release by both axons and astrocytes, triggers calcium currents through P2Y and P2X receptors (Hamilton et al., 2010). Yet, depolarizations and calcium transients are mostly described as very local events that take place at the cell processes, where synapses are mostly found, and could therefore influence spatially restricted functions such as local protein synthesis, motility, or secretion (see also above, Kirby et al., 2006; Tanaka et al., 2009; Haberlandt et al., 2011; Wake et al., 2011; Hughes et al., 2013).

What is the functional significance of neuronal inputs? Since NG2+p do not appear able to transmit electrical signals to other cells, information derived from neuronal activity is likely to instruct functions specific to these progenitors. Several reports showed that neurotransmitters can affect proliferation and migration of NG2+p *in vitro* (Luyt et al., 2007; Gallo et al., 2008; Tong et al., 2009). Other studies related alterations in circuit activities at adult ages (including motor activity, sleep-wake cycles, experimental spreading depression, or enriched environment) to modulation of NG2+p proliferation and maturation (Ehninger et al., 2011; Simon et al., 2011; Tamura et al., 2012; Bellesi et al., 2013). However, these findings, which appeared somewhat contradictory, only established a rather aspecific link between neuronal activity and NG2+p behaviors. In a recent report Mangin et al. (2012) addressed this issue more directly and found that sensory stimuli from the whisker pad regulate NG2+p number and distribution in the neonatal barrel cortex by negatively affecting cell proliferation. These data are in keeping with an inhibitory role of glutamatergic inputs on NG2 cell amplification and suggest that different inputs levels would result in proliferation-mediated accumulation of NG2+p at sites of relatively low electric activity. Such accumulations could then specifically predispose cells to start myelination (Mangin et al., 2012), as the achievement of a critical density is one of the key factors for myelin formation (Rosenberg et al., 2008). Electrical activity itself could likely further support the progression of postmitotic progenitors along the lineage, as it has long been known to be a myelination promoter (Demerens et al., 1996; Stevens et al., 2002; Lundgaard et al., 2013). Thus, the response of NG2+p to neuronal activity appears crucial to regulate their number and engagement in myelination during development, thereby contributing to structure the CNS architecture. Similarly, it could underlie myelin refinements related to learning and memory during adulthood. Yet, how these findings apply to the adult CNS and whether the large abundance of NG2+p present at adult stages is exclusively required to sustain the low grade of myelin turnover so far detected and/or experience-related circuit modulations remain to be assessed. Indeed, the occurrence of myelin remodeling appears rather low in the adult CNS (Zatorre et al., 2012; Young et al., 2013) and at least in the gray matter the overall rate of NG2+p proliferation does not differ in myelin rich vs. myelin low territories (our unpublished observations) thus showing to be unrelated to myelin turnover. Further, exploiting the high metabolic charge of electric activity solely to tonically limit NG2+p proliferation appears poorly efficient, particularly in light of data showing that homotypic density cues play a major role in the regulation of NG2+p proliferation at adult stages (Hughes et al., 2013). Moreover, the observation that an important part of NG2+ clones in the gray matter do not generate oligodendrocytes in the adult brain (Levison et al., 1999; Zhu et al., 2011) raises the question as to whether NG2+p responses to neuronal inputs may include functions other than proliferation control or lineage progression.

## Ng2+p functions beyond myelination?

Recently discovered features of NG2+p point to additional neuromodulatory and neuroprotective actions of this population. Some of these traits appear specifically related to the particular capability of NG2+p to sense and respond to electrical activity. Others, including the expression of growth factors, morphogens, cytokines, chemokines, and extracellular matrix (ECM) components, can instead be viewed as properties inherent in the progenitor nature of these cells, and may well correspond to the reparative bystander actions that germinal neural progenitors exert either *in situ* or upon grafting in the lesioned CNS (Martino and Pluchino, 2006; Butti et al., 2012).

Of note, evidence that NG2+p express synaptophysin suggests they may be capable of regulated secretion and bidirectional communication with astrocytes and neurons (Wigley and Butt, 2009). Of relevance in this context, Maldonado et al. (2013) have shown that during postnatal maturation NG2+p become progressively more sensitive to extracellular potassium increases generated by action potentials thanks to upregulation of Kir4.1 inward rectifying potassium channels. These channels mediate inward currents in conditions of high potassium and constitute one of the mechanisms through which astrocytes perform potassium buffering in the extracellular space, which is a requisite for correct neuronal transmission and excitability. In adult NG2+p, besides contributing to set the resting membrane potential, these channels may help with removing high extracellular potassium and regulating neuronal functions at sites of close appositions to neurons (see above) (Maldonado et al., 2013). This could be a NG2+p specific mechanisms occurring upon single neuron firing, which may remain undetected by astrocytes (Maldonado et al., 2013).

NG2+p are also known to express several ECM components such as tenascins, versicans, neurocans, phosphacan, hyaluronan and even hyaluronan, and proteoglycan link protein 1 (HPLN1), which act as synaptic stabilizers by anchoring the neurotransmitter receptors to the cytoskeleton and contribute to perineuronal nets formation (Butt et al., 2005; Sim et al., 2009; see also Sim and Goldman, 2013). Given the intimate association of NG2 processes with neuronal pre- and post-synaptic elements and that neighboring pairs of NG2+p and neurons receive synaptic contacts from the same neurons (Bergles et al., 2000; Lin et al., 2005; Mangin et al., 2008), it can be hypothesized that NG2+p monitor neuron-neuron synapses and, depending on synchronization levels, act on synaptic stabilization and network organization by modifying the perisynaptic microenvironment. Interestingly, human NG2+p also show enriched levels of thrombospondin 2 (Sim et al., 2006), which is known as a synaptogenic cue released by immature astrocytes (Christopherson et al., 2005). Seminal studies demonstrating the role of astroglia in synaptic strengthening and formation also attributed a relevant effect to cells of the oligodendroglial lineage (Pfrieger and Barres, 1997). However, these data should be re-considered in light of the need to clarify the purity of cells and actual maturation stages. Yet, in line with an early function in circuit formation, acute deletion of cycling oligodendroglial cells have been reported to induce rapid changes in the expression of molecules involved in synaptic plasticity, axon growth and guidance in the cerebellum at birth, indicative of fast activation of remodeling mechanisms (Doretto et al., 2011). Previous experiments at later developmental stages have shown that myelin formation shapes cerebellar connections by removing exuberant collateral branches of Purkinje neurons (Gianola et al., 2003). These results suggest that cycling NG2+p participate in shaping cerebellar circuits well before myelination starts.

Neuroprotective mechanisms have been also proposed to be activated in NG2+ cells upon lesion. NG2+p provide a stereotyped response to injury, unrelated to the extent of myelin loss and of their own damage, which includes a precocious activation of proliferation and hypertrophy and is mediated by inflammatory and danger related signals (Levine et al., 2001; Nielsen et al., 2006). The sensitivity of these cells to changes in nerve conduction and neurotransmission could also influence NG2+p reactivity. Tanaka et al. (2009) reported that GABA-receptor mediated excitation in NG2+p after ischemic stroke increases brain-derived neurotrophic factor (BDNF) production, which was instead blocked by inhibition of GABA-mediated depolarization. The authors further hypothesize that NG2+p-derived BDNF participate in post-stroke reparative mechanisms. Given the well-known actions of BDNF in promoting synaptic transmission, plasticity, and growth (Lu et al., 2013), these speculations could be extended to include modulatory actions of neuronal functions in physiological conditions. Along this line, reactive NG2+p responding to depolarizing waves induced by experimental cortical spreading depression were reported to upregulate the peptide galanin and proposed to release it to receptor positive cortical neurons with the purpose of limiting excitotoxic damage (Shen et al., 2003). In a recent *in vitro* study Sypecka and Sarnowska (2013) provided first evidence in support of a pro-survival action of NG2+p on the injured nervous tissue. In co-cultures of primary NG2+p with organotypic hippocampal slices subjected to oxygen-glucose deprivation, the authors observed a significant rescue of neuronal viability and identify BDNF, interleukin-10, stem cell factor (SCF) as agents through which NG2+p perform immunomodulatory and protective functions in their experimental setting. Accordingly, NG2+p derived from human embryonic stem cells express transforming growth factor (TGF) β 2, a potent inhibitor of inflammation, midkine, and activine A, two neurosupportive factors that are highly upregulated early after injury (Munz et al., 2001; Zhang et al., 2006; Yoshida et al., 2014). NG2+p cells could therefore share the capability of mature oligodendrocytes to produce neurotrophic factors, influence adjacent cells (Wilkins et al., 2001; Dai et al., 2003) and especially neurons in physiology and conditions of cell stress (Lee et al., 2012; Frühbeis et al., 2013). Further, in amyloidosis models it has been reported that NG2+p internalize and degrade β-amyloid1-42 by autophagy (Li et al., 2013). Thus, together with astrocytes and microglia, NG2+ glia could also participate in the clearance of amyloid as part of their reactive response (Boda et al., 2011; Behrendt et al., 2013).

The potential reparative and supportive actions may be specifically triggered by NG2+p reactivity or be already present in resting conditions and become amplified as a consequence of the NG2+p widespread cytogenic response to damage. However, these considerations remain speculative, because a detailed and comprehensive examination of NG2+p changes upon lesion is not available. What is instead better assessed is the contribution of NG2+p to scar formation and the inhibitory role of chondroitin sulfate proteoglycans—including NG2—in axon remodeling and regrowth of transected axons (Galtrey and Fawcett, 2007), recently accompanied by evidence for conduction blockade exerted by NG2 at nodes of Ranvier after spinal cord transection (Petrosyan et al., 2013). While these inhibitory effects are mostly seen as detrimental for circuit rewiring, they could be part of a mechanism necessary to contain excitoxicity and damage extension, as formerly shown for astrocytes (Buffo et al., 2010). Further, actions restricting plasticity may be compensated by an increment in the availability of prosurvival factors determined by both NG2+p reactivity and proliferation.

## Concluding remarks

Intense research over the last decades has revealed key insights into NG2+p physiopathology related to myelinogenesis. Nevertheless, fundamental aspects of NG2+p biology remain undetermined. For example, while NG2+p are known to respond to signals produced by neurons, astrocytes, and microglia, whether this communication is reciprocal and how it occurs is substantially neglected. NG2+p-derived signaling may include morphogenic, neuromodulatory, and neuroprotective factors whose elucidation may also have therapeutic implications for the implementation of the endogenous reparative potential of injured CNS. *In vivo* approaches aimed at selectively and timely ablating NG2+p in the CNS, together with the identification of active paracrine/juxtacrine factors produced by NG2+p, will be highly instrumental to address these issues, especially to understand how much it is crucial to maintain such a high number of cells to sustain myelin turnover and plasticity in the adult CNS and whether any relevant alteration in neuronal functions occurs in the absence of NG2+p in the intact or injured CNS.

### Conflict of interest statement

The authors declare that the research was conducted in the absence of any commercial or financial relationships that could be construed as a potential conflict of interest.
